# Increased sensitivity to cocaine-induced analgesia in Spontaneously Hypertensive Rats (SHR)

**DOI:** 10.1186/1744-9081-3-9

**Published:** 2007-02-13

**Authors:** Fabrício A Pamplona, Leandro F Vendruscolo, Reinaldo N Takahashi

**Affiliations:** 1 Departamento de Farmacologia, Centro de Ciências Biológicas, Universidade Federal de Santa Catarina, Florianópolis, SC, Brazil

## Abstract

This study examined the analgesic effect of cocaine in Spontaneously Hypertensive Rats (SHR), which are considered a suitable model for the study of attention deficit hyperactivity disorder (ADHD), and in Wistar (WIS) rats of both sexes using the hot-plate test. In addition, we tested whether habituation to the unheated hot-plate apparatus, that "normalizes" the basal hypoalgesic phenotype of SHR, alters the subsequent cocaine-induced analgesia (CIA) in this strain. SHR of both sexes were hypoalgesic compared to WIS rats in the hot-plate test and showed higher sensitivity to CIA. Habituation to the unheated hot-plate reduced the basal nociceptive latency of SHR, suggesting cognitive/emotional modulation of pain in this strain, but did not alter the magnitude of CIA. The present study shows increased sensitivity to CIA in SHR, which may be related to abnormalities in the mesocorticolimbic dopaminergic system. Further studies using SHR strain may reveal new information on the neurobiological mechanisms underlying ADHD and its co-morbidity with drug addiction.

## Findings

Pain is a complex and subjective experience that involves the transduction of noxious stimuli by nociceptive fibers, but also cognitive and emotional aspects [1]. For instance, human studies indicate that pain is perceived as less intense when individuals are distracted from the pain [2]. Gender and genetic differences also influence the pain perception and a number of animal models have been used to study the influence of these factors on nociception [3]. The Spontaneously Hypertensive Rats (SHR) show abnormal nociceptive reactivity in several nociceptive tests [4-8]. In the hot-plate test, SHR are hypoalgesic when compared to rats of other strains [4,5,7,8], but they show normal properties of nociceptive fibers [9]. We have recently reported that hypoalgesia was no longer observed in SHR rats after habituation to the unheated hot-plate apparatus, suggesting that their hypoalgesic phenotype may involve cognitive processes (e.g. distraction) [8]. This is consistent with the fact that SHR have been considered an animal model for the study of attention deficit hyperactivity disorder (ADHD), since they show inattention and impulsivity/hyperactivity [10,11]. It remains to be clarified whether this characteristic of SHR interferes with the analgesic properties of drugs.

Alterations in the dopaminergic system in ADHD patients as well as in SHR have been identified [10,11]. Methylphenidate, the first-choice treatment for ADHD, is known to block dopamine (DA) uptake by brain DA transporters in a similar way to potent psychostimulants like cocaine and amphetamine [12]. In addition, the mesocorticolimbic dopaminergic system is one of the main neurochemical pathways involved in the interface between pain, cognition and emotionality [13]. Systemic administration of DA re-uptake blockers induces analgesia in rodents, probably by acting on brain dopaminergic pathways [14,15].

The first aim of this study was to examine the effects of cocaine on nociceptive responses in the SHR and in the outbred Wistar (WIS) rat strain (representing a "normal" genetically heterogenic population) using the hot-plate test. In order to evaluate the contribution of cognitive/emotional processes in the analgesic effect of cocaine, another objective of our study was to evaluate whether habituation to the unheated hot-plate apparatus, that "normalizes" the basal nociception of SHR, alters the subsequent cocaine-induced analgesia (CIA) in this strain. Animals of both sexes were included in this study because there is a considerable amount of evidence for quantitative and qualitative sex differences in nociceptive-related behaviors [3].

Adult (12 weeks old) SHR and WIS rats from our own colonies were used [8]. The weight of the animals was 230–310 g for males and 150–210 g for females. They were housed collectively in plastic cages (5–6/cage), under controlled temperature (23 ± 2°C) with a 12-h light/dark cycle (lights on at 07:00) with free access to rat chow and tap water. All experiments were carried out during the light phase of the cycle.

The animals were injected with cocaine (20 mg/kg, Merck^®^) dissolved in physiological solution or an equivalent amount of vehicle (2 ml/kg) via intraperitoneal (i.p.) route 15 min before the nociceptive tests. The dose of cocaine was selected based on a pilot study and a previous report [14]. All procedures performed complied with the "Principles of laboratory animal care" from NIH.

The hot-plate (Ugo Basile, model-DS37) was maintained at 52.2 ± 0.5°C following a previously reported procedure [8]. Briefly, the animals were placed in a glass cylinder of 24-cm diameter on the heated metal surface, and the time between placement and hind paw licking or jumping (whichever occurred first) was recorded as nociceptive latency. A 70-s cut-off was established to prevent tissue damage. The procedure of the habituation to the hot-plate apparatus has been described elsewhere [8]. SHR were submitted to five sessions of 90-s exposure (at 10-min inter-trial intervals) to the unheated hot-plate apparatus. Another group of rats remained undisturbed in their home cages and served as non-habituated animals (test naive rats). One hour after the last habituation session, habituated and non-habituated rats were injected with cocaine as previously described and tested on the hot-plate. Because cocaine-induced analgesia was of similar intensity in both genders, this experiment was carried out only with female rats due to their greater availability in our laboratory.

The results were expressed as the latency (s) to nociception or the percentage of maximum possible effect (% MPE) defined by the following equation:

%MPE=post-drug latency−basal latencycut-off−basal latency×100
 MathType@MTEF@5@5@+=feaafiart1ev1aaatCvAUfKttLearuWrP9MDH5MBPbIqV92AaeXatLxBI9gBaebbnrfifHhDYfgasaacH8akY=wiFfYdH8Gipec8Eeeu0xXdbba9frFj0=OqFfea0dXdd9vqai=hGuQ8kuc9pgc9s8qqaq=dirpe0xb9q8qiLsFr0=vr0=vr0dc8meaabaqaciaacaGaaeqabaqabeGadaaakeaacqGGLaqjcqqGnbqtcqqGqbaucqqGfbqrcqGH9aqpdaWcaaqaaiabbchaWjabb+gaVjabbohaZjabbsha0jabb2caTiabbsgaKjabbkhaYjabbwha1jabbEgaNjabbccaGiabbYgaSjabbggaHjabbsha0jabbwgaLjabb6gaUjabbogaJjabbMha5jabgkHiTiabbkgaIjabbggaHjabbohaZjabbggaHjabbYgaSjabbccaGiabbYgaSjabbggaHjabbsha0jabbwgaLjabb6gaUjabbogaJjabbMha5bqaaiabbogaJjabbwha1jabbsha0jabb2caTiabb+gaVjabbAgaMjabbAgaMjabgkHiTiabbkgaIjabbggaHjabbohaZjabbggaHjabbYgaSjabbccaGiabbYgaSjabbggaHjabbsha0jabbwgaLjabb6gaUjabbogaJjabbMha5baacqGHxdaTcqaIXaqmcqaIWaamcqaIWaamaaa@7A26@

Statistical analysis was performed using one-, two- or three-way ANOVA with condition (habituated and non-habituated) or strain and gender as factors. Repeated measure was included as an analysis factor for comparisons of nociceptive latencies before and after drug treatment. The Newman-Keuls test was used for post-hoc comparisons. The accepted level of significance was p < 0.05.

**Figure 1:**
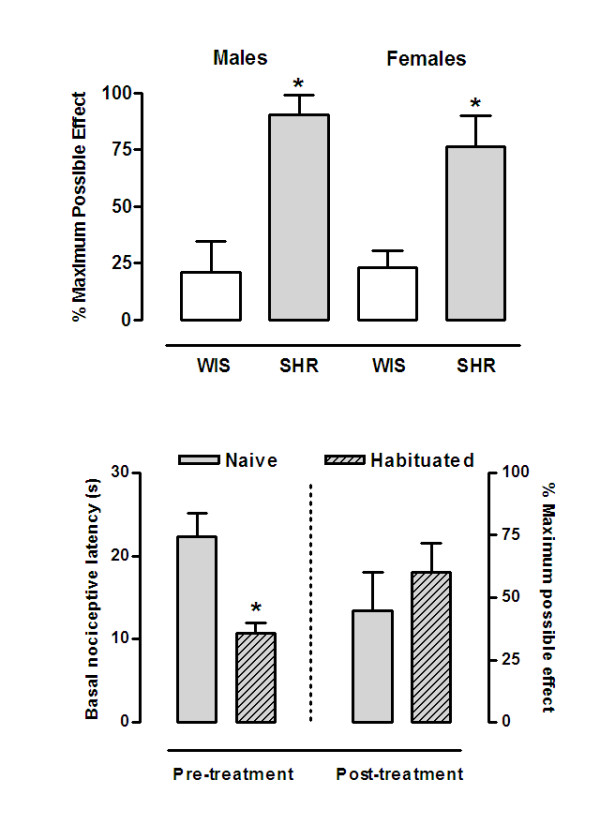
Cocaine-induced analgesia in Wistar and Spontaneously Hypertensive Rats in the hot-plate test. Male and female rats of Wistar (WIS) and Spontaneously Hypertensive Rats (SHR) strains were evaluated in the hot-plate test 15 min after i.p. injection of cocaine (20 mg/kg) (upper panel). SHR rats were habituated to the unheated hot-plate apparatus and 1 h after evaluated for CIA in the hot-plate test (lower panel). Data are presented as nociceptive latencies (s) or the percentage of maximum possible effects (%MPE). * p < 0.05 vs WIS rats of the corresponding gender (upper panel) or vs SHR naive rats (lower panel) (Newman-Keuls post hoc test).

The three-way ANOVA (strain, gender and repeated measure) for the nociceptive latencies in the hot-plate test revealed effect for strain [F_(1,24) _= 50.45; p < 0.0001], repeated measure [F_(1,24) _= 62.94; p < 0.0001] and for strain vs repeated measure interaction [F_(1,24) _= 18.69; p < 0.001]. SHR of both sexes (males: 23.7 ± 2.4 s, females: 26.7 ± 2.3 s) showed higher basal latencies to nociception (i.e. hypoalgesia) than WIS rats (males: 15.8 ± 1.1 s, females: 11.0 ± 1.1 s). The analgesic effect of cocaine in the hot-plate test displayed by SHR and WIS rats of both sexes is illustrated in Figure 1 (upper panel). The two-way ANOVA for the % MPE revealed an overall effect of strain [F_(1,24) _= 29.59; p < 0.0001], indicating that male and female SHR showed higher CIA compared to their WIS counterparts (p < 0.05).

Figure 1 (lower panel) illustrates the basal hot-plate latencies and % MPE of habituated and nonhabituated SHR. One-way ANOVA (condition) for the basal latencies revealed that habituated SHR showed lower nociceptive latencies compared to test naive rats [F_(1,11) _= 18.18; p < 0.005]. Habituated and nonhabituated rats displayed analgesic effects of cocaine [F_(1,11) _= 27.46; p < 0.001]. However, one-way ANOVA (condition) for the % MPE indicated that CIA was similar in these groups [F_(1,11) _= 0.63; p = 0.44].

This study provides evidence of increased sensitivity to cocaine in terms of analgesia in the SHR strain when compared to the WIS strain in the hot-plate test. Moreover, habituation to the unheated hot-plate reduced the basal nociceptive latency of SHR without altering the magnitude of the analgesic effect of cocaine.

SHR are hypoalgesic compared to Wistar, Wistar-Kyoto, Sprague-Dawley and Lewis rats [4-8,16]. Thus, the results here presented for basal nociception are in agreement with the aforementioned findings. As pointed out by Taylor et al., the exaggerated fight-or-flight stress responses and sympathetic activation in SHR may be related to their abnormal nociceptive phenotype [7]. Several studies have shown that stress can cause intense analgesia [8,17]; however, we have reported that when a more severe stressor was employed (forced swimming in cold water), SHR were less vulnerable to stress-induced analgesia compared to Lewis and WIS rats [8]. This apparent discrepancy regarding an "analgesic" effect produced by exposure in a novel environment and the reduced impact of stressful situations in SHR suggest that this hypoalgesic phenotype is more likely to be a result of distraction of the SHR from the nociceptive stimulus (heated plate) rather than reflecting stress-induced analgesia. Confirming our previous study [8], habituation of SHR to the unheated hot-plate resulted in a striking reduction of the basal nociceptive latency. These findings, taken together with the fact that the SHR strain is considered a suitable model of ADHD [10,11], emphasizes that SHR hypoalgesia probably involves cognitive/emotional processes. Finally, several studies suggest that the nociception of SHR is not associated with their inherited hypertensive trait [4,7].

SHR show increased sensitivity to CIA compared to WIS rats. Moreover, habituation to the hot-plate that "normalizes" the hypoalgesic phenotype of SHR did not influence CIA intensity. There are findings in support of an increased sensitivity of SHR to other behavioral effects of psychostimulants. For instance, SHR show increased susceptibility to convulsions induced by cocaine [18] and increased psychomotor stimulation induced by amphetamine [19] or methylphenidate [20]. (Although see [21,22]). Alterations in DA neurotransmission have been extensively described in the SHR strain, including reduced release of DA in the prefrontal cortex, nucleus accumbens (NAcc) and striatum [23], decreased DA turnover in the substantia nigra, ventral tegmental area and frontal cortex [23], reduced DA vesicular storage [24] and increased density of the D1/D5 receptors in the anterior forebrain [25]. Interestingly, stimulus-evoked release of DA was lower in the striatum of SHR [26], although d-amphetamine evoked greater release of DA in the prefrontal cortex, NAcc and striatum of SHR compared to Wistar-Kyoto rats [24].

In summary, SHR have increased sensitivity to behavioral and neurochemical effects of psychostimulants, which may be related to abnormalities in the mesocorticolimbic dopaminergic system. Because hyperlocomotion and analgesia share a common neural substrate with the rewarding effects of drugs of abuse [13], it is possible to suggest that the enhanced behavioral effects of cocaine in SHR could reflect the higher preference of SHR for drugs of abuse [27]. Thus, further studies using SHR strain may reveal new information on the neurobiological mechanisms underlying ADHD and its co-morbidity with drug addiction.

## Competing interests

The author(s) declare that they have no competing interests.

## Authors' contributions

FAP carried out the data collection, designed the study and wrote the manuscript. LFV and RNT participated in the data analyses, interpretation of data and elaboration of the manuscript. All authors read and approved the final manuscript.
